# A Novel, Stable, Estradiol-Stimulating, Osteogenic Yam Protein with Potential for the Treatment of Menopausal Syndrome

**DOI:** 10.1038/srep10179

**Published:** 2015-07-10

**Authors:** Kam Lok Wong, Yau Ming Lai, Ka Wan Li, Kai Fai Lee, Tzi Bun Ng, Ho Pan Cheung, Yan Bo Zhang, Lixing Lao, Ricky Ngok-Shun Wong, Pang Chui Shaw, Jack Ho Wong, Zhang-Jin Zhang, Jenny Ka Wing Lam, YE Wencai, Stephen Cho Wing Sze

**Affiliations:** 1 School of Chinese Medicine, Li Ka Shing Faculty of Medicine, The University of Hong Kong, Pokfulam, Hong Kong SAR, China; 2 Department of Health Technology and Informatics, The Hong Kong Polytechnic University, Hung Hom, Hong Kong SAR, China; 3 Department of Molecular and Cellular Neurobiology, VU University Amsterdam, Amsterdam, The Netherlands; 4 Department of Obstetrics and Gynaecology, Li Ka Shing Faculty of Medicine, The University of Hong Kong, Pokfulam, Hong Kong SAR, China; 5 School of Biomedical Sciences, Faculty of Medicine, The Chinese University of Hong Kong, Shatin, N.T., Hong Kong SAR, China; 6 Department of Biology, Faculty of Science, Hong Kong Baptist University, Hong Kong SAR, China; 7 School of Life Sciences and Centre for Protein Science and Crystallography, Faculty of Science, The Chinese University of Hong Kong, Shatin, N.T., Hong Kong SAR, China; 8 Department of Pharmacology & Pharmacy, Li Ka Shing Faculty of Medicine, The University of Hong Kong, Pokfulam, Hong Kong SAR, China; 9 Institute of Traditional Chinese Medicine and Natural Products, Jinan University, Guangzhou, Guangdong Province, China

## Abstract

A novel protein, designated as DOI, isolated from the Chinese yam (*Dioscorea opposita* Thunb.) could be the first protein drug for the treatment of menopausal syndrome and an alternative to hormone replacement therapy (HRT), which is known to have undesirable side effects. DOI is an acid- and thermo-stable protein with a distinctive N-terminal sequence Gly-Ile-Gly-Lys-Ile-Thr-Thr-Tyr-Trp-Gly-Gln-Tyr-Ser-Asp-Glu-Pro-Ser-Leu-Thr-Glu. DOI was found to stimulate estradiol biosynthesis in rat ovarian granulosa cells; induce estradiol and progesterone secretion in 16- to 18-month-old female Sprague Dawley rats by upregulating expressions of follicle-stimulating hormone receptor and ovarian aromatase; counteract the progression of osteoporosis and augment bone mineral density; and improve cognitive functioning by upregulating protein expressions of brain-derived neurotrophic factor and TrkB receptors in the prefrontal cortex. Furthermore, DOI did not stimulate the proliferation of breast cancer and ovarian cancer cells, which suggest it could be a more efficacious and safer alternative to HRT.

Menopausal syndrome refers to the symptoms experienced by women during menopause, such as hot flushes, mood disorders, night sweats, depression, nervous tension, and insomnia^1^,^2^. Menopause also puts women at increased risk of cardiovascular disease and osteoporosis. The total worldwide population of postmenopausal women was 476 million in 1990 as reported by the World Health Organization, and 477 million in 1998 as reported by the North American Menopausal Society. By 2030, it is predicted that this population will reach 1200 million^3^. The current approach for relieving menopausal syndrome is hormone replacement therapy (HRT), which restores the endogenous estrogen level using supplementary exogenous estrogen with or without progestin^4^,^5^. Estradiol and conjugated estrogens have been proven effective in the treatment of vasomotor symptoms, urogenital atrophy, and irregular menstrual bleeding^6^,^7^,^8^,^9^,^10^. However, HRT can have side effects, which need to be taken into consideration. The risk of endometrial cancer has been shown to be associated with HRT in a dose-dependent manner, and even treatments using a low dose of a weak estrogen may induce endometrial cancer^11^. HRT is also believed to be associated with breast cancer, ovarian cancer^12^, venous thromboembolism, and stroke^13^. Although formulations of HRT have shown improvements in recent years, its side effects require further evaluation in clinical studies. Therefore, safe and effective therapeutics for menopausal syndrome are needed. Tissue-specific estradiol-stimulating components and potential drug candidates for the treatment of menopausal syndrome would be desirable as safer alternatives to HRT.

Yam tubers, belonging to the monocotyledonous *Dioscorea* genus, are a major staple food crop in tropical and subtropical regions, with an annual world production of around 58.1 million tons in 2012^14^. The *Dioscorea* family has more than 600 species found throughout the world and 93 are found in China, of which 13 are edible and are widely consumed as a staple food or tonic food^15^. Rhizoma of *Dioscorea opposita* (*Shanyao*) has been included in the Pharmacopoeia of the People’s Republic of China as a Chinese herbal medicine. It is by far the most commonly used *Dioscorea* species for improving female health and for regulating menstruation^16^. The chemical composition and pharmacology of many *Dioscorea* species have been extensively studied. Dioscin (glycoside form of diosgenin) is a type of saponin isolated from the n-butanol fraction of extracts from *Dioscorea batatas* DECNE, which was found to induce the release of growth hormones from rat pituitary cells and elevate plasma levels in rats^17^. Diosgenin displays impressive pharmacological properties^18^ and is structurally similar to the precursor of steroid hormones^19^. Diosgenin can be converted to steroid hormones by industrial processes^20^,^21^, but cannot be biochemically transformed into steroid hormones by the human body^19^. Interestingly, previous clinical investigations indicated that *Dioscorea* extract was effective in mitigating menopausal syndrome by elevating serum estrogen levels^22^. Therefore, the estrogenic ability exhibited by *Dioscorea* tubers needs to be identified and further investigated to uncover the mechanisms involved. Our pilot study found that total protein extracts of *Dioscorea opposita* exhibited stimulating effects on estrogen biosynthesis in rat ovarian granulosa cells.

In this study, we aimed to isolate and characterize the bioactive protein, designated DOI, from *Dioscorea opposita*. The DOI protein of *Dioscorea opposita* was extracted and purified by fast protein liquid chromatography (FPLC). DOI was chemically characterized by SDS-PAGE and silver staining, and by N-terminal and partial amino acid sequencing. DOI was biologically characterized by *in vitro* estrogenic assay; acid, alkali and thermal stability tests; viability assays in ovarian granulosa cells, cancer cells and immune cells to evaluate the cancer risk; and chitinase activity assay. Results obtained from the biological characterization demonstrated that DOI had potent estrogen-stimulating activity. The efficacy of DOI for treating physiological states characterized by estrogen deficiency (e.g., menopause) was evaluated by determining the serum estrogen and progesterone levels, brain derived neurotrophic factor (BDNF) levels, and bone mineral density in a menopausal rat model treated with DOI. We also studied the molecular actions of DOI in relation to ovarian steroidogenesis including ovarian follicle-stimulating hormone receptor (FSHR), protein kinase A, B, C (PKA, PKB, PKC) and aromatase, and cognitive functions including BDNF and TrkB gp145.

## Results

### DOI protein extraction and column purification

**Figure 1:**
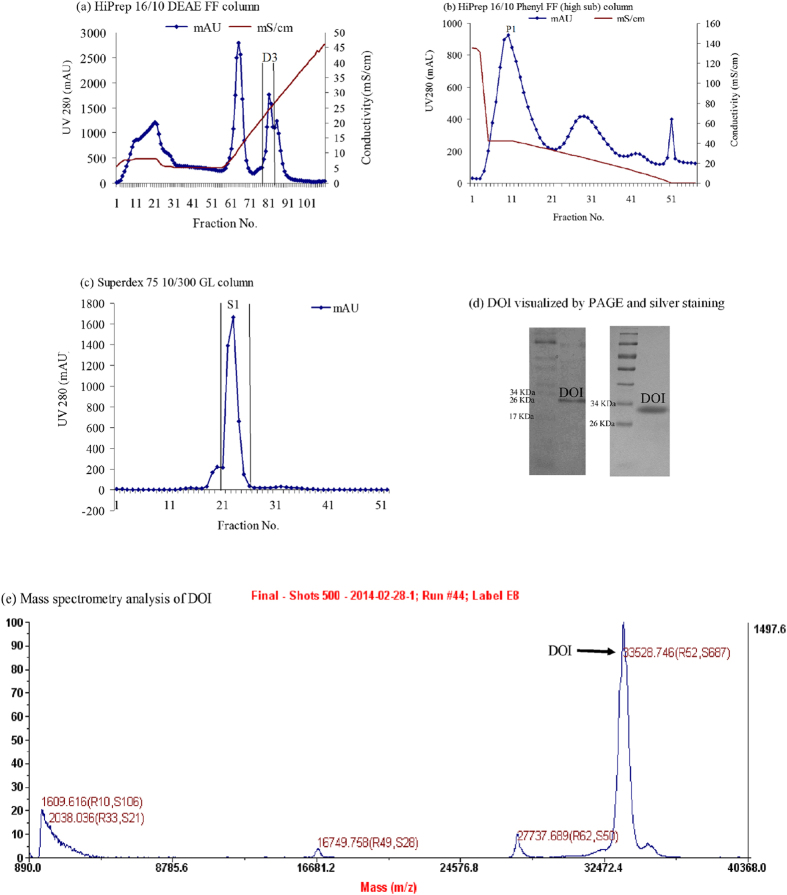
(**a**) Purification of DOI on a HiPrep 16/10 DEAE FF column. (**b**) Fraction D3 was further purified on a HiPrep 16/10 Phenyl FF (high sub) column. (**c**) Fraction P1 was further purified on a Superdex 75 10/300 GL column and fraction S1 contained the DOI protein. (**d**) 15% Native PAGE of DOI (left) and 15% SDS-PAGE of DOI (right). The DOI protein was visualized by silver staining. (**e**) Size measurement of DOI by mass spectrometry.

Crude protein extracts were obtained from the Chinese yam, *Dioscorea opposita* Thunb. The protein preparations were separated by chromatography on a HiPrep 16/10 DEAE FF column using an AKTA Purifier Fast Protein Liquid Chromatography (FPLC) system (Fig. 1a). Fraction D3 (third peak) had estrogen-stimulating activity and was collected and dialyzed. It was purified on a HiPrep 16/10 Phenyl FF (high sub) column by FPLC. Active fraction P1 (Fig. 1b) was dialyzed and further purified on a Superdex 75 10/300 GL column by FPLC. The purified active protein (fraction S1), designated as DOI, was obtained at a yield of 0.36% of the total soluble proteins extracted from *Dioscorea opposita* Thunb. The purification steps are summarized in Supplementary Table S1.

### Chemical characterization of DOI

The high purity of the isolated DOI protein was verified and DOI was visualized as a single band in 15% SDS-PAGE and silver staining (Fig. 1d). The molecular weight of DOI was 32.5 kDa as measured by size-exclusion chromatography and SDS-PAGE (Supplementary Table S2), and was 33.5 kDa as measured by mass spectrometry (Fig. 1e). The N-terminal sequence of DOI was GIGKITTYWGQYSDEPSLTE as determined by Edman degradation. A partial internal amino acid sequence of DOI was KSFYTR**SNFLEAVSAYPGFGTK**REIAAYFAHVTH**GPMQLSWNYNYIDAGKELHFDGLNDPDIVGRDPIISFKTSLWFWIRKGVQYVILDPNQGFGATIR**IINGGQECDGHNTAQMMARVGYYQEYCAQ as determined by mass spectrometry.

**Figure 2:**
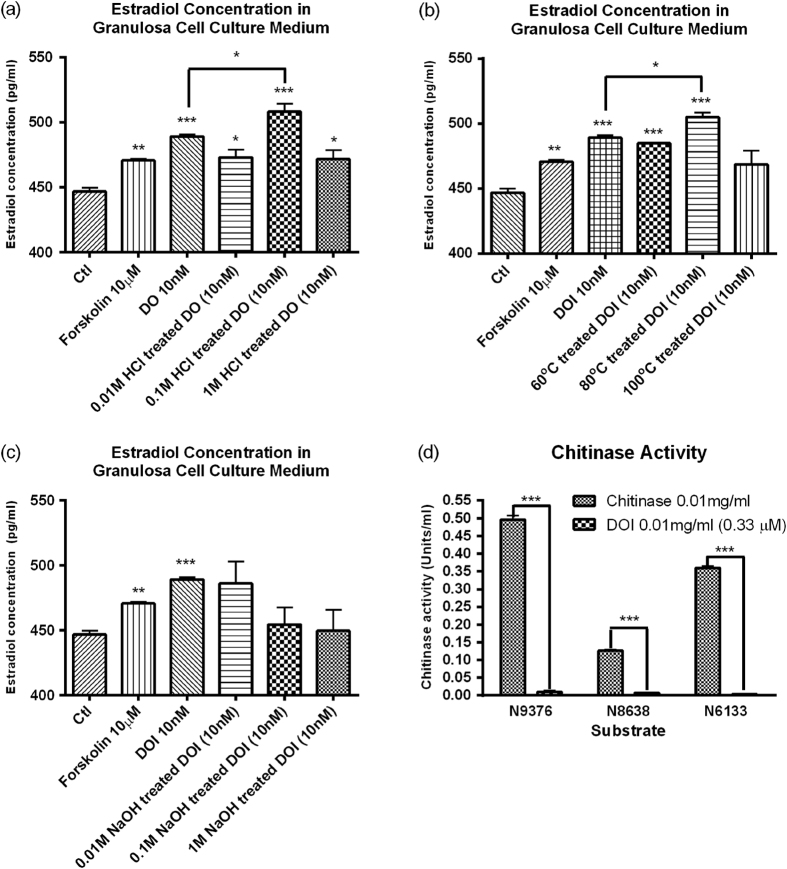
Estrogen-stimulating activity of (**a**) HCl-, (**b**) NaOH-, (**c**) heat-treated DOI on granulosa cells. Results are expressed as means ± SEM (n = 3). *p < 0.05, **p < 0.01, ***p < 0.001 compared with the control group (un-paired *t*-test). (**d**) Chitinase activity of DOI. Results are expressed as means ± SEM (n = 3). **p < 0.01, ***p < 0.001 compared with the positive chitinase control (un-paired *t*-test). N9376: 4-Nitrophenyl N-acetyl-β-D-glucosaminide used to detect exochitinase activity (β-N-acetylglucosaminidase activity); N8638: 4-Nitrophenyl β-D-N, N’, N”triacetylchitotriose used to detect endochitinase activity; and N6133: 4-Nitrophenyl N, N′-diacetyl-β-D-chitobioside used to detect exochitinase activity (chitobiosidase activity).

BLAST analysis of the N-terminal sequence of DOI in NCBI showed a high E-value (>10^−3^) that indicated it was a novel protein. The results showed DOI was a member of the chitinase-like superfamily (Supplementary Table S3). BLAST analysis of the partial amino acid sequence of DOI revealed that it had high homology with the 27.9-kDa chitinase (AAB23692.1) from *Dioscorea japonica*^23^ and the 31.4-kDa chitinase (BAC56863.1) from *Dioscorea oppositifolia*^24^. Mass spectrometry analysis of tryptic peptides from DOI identified 10 peptides by MaxQuant that are matched to the acidic endochitinase from Dioscorea japonica. The identified peptides are highlighted in bold in the partial sequence of acidic endochitinase. Our isolated DOI protein had a comparable molecular weight of 33.5 kDa as measured by mass spectrometry (Fig. 1e). The N-terminal sequence of the isolated DOI showed differences from the 31.4-kDa BAC56863.1 chitinase from *Dioscorea oppositifolia*^24^. In addition, the chitinase activity of DOI was negligible using three different substrates, 4-nitrophenyl N,N′-diacetyl-β-D-chitobioside (N6133), 4-nitrophenylN-acetyl-β-D-glucosaminide (9376) and 4-nitrophenyl β-D-N,N′,N′′-triacetylchitotriose (N8638), in the chitinase assay (Fig. 2d). These differences may be due to alternative splicing and post-translational modifications. This data suggests that DOI might be a novel protein.

### Biological characterization of DOI

The DOI protein did not have any hemagglutinating activity, which suggested it was not a lectin. DOI (1 to 10 nM) displayed estrogen-stimulating activity in rat ovarian granulosa cells but not at concentrations of 100 nM (Fig. 2). DOI (10 nM) showed acid stability as its estrogen-stimulating activity remained intact after treatment with HCl (0.01–1 M) (Fig. 2a) and also showed themo-stabilty at 80 °C (Fig. 2c), but it did not show alkali stability in NaOH (0.01–1 M) (Fig. 2b). As mentioned, DOI did not show chitinase activity compared with the positive chitinase control (Fig. 2d).

**Figure 3:**
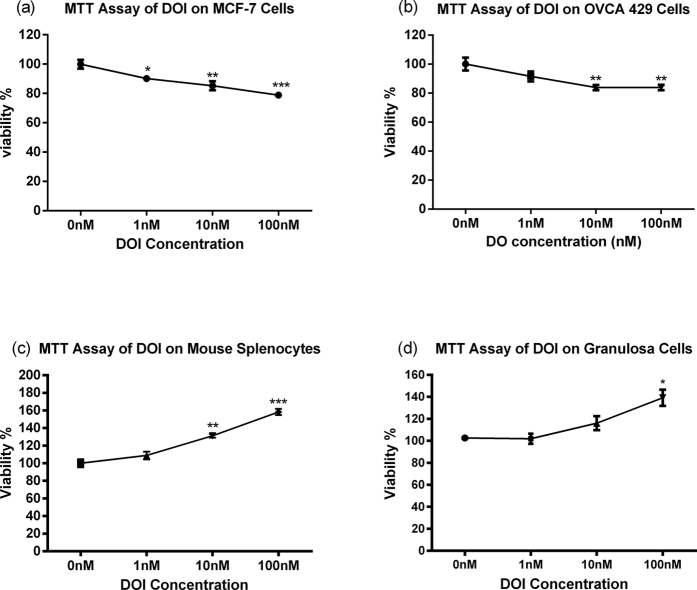
Viability of (**a**) MCF-7 breast cancer cells, (**b**) OVCA-429 ovarian cancer cells, (**c**) mouse splenocytes, and (**d**) ovarian granulosa cells after treatment with DOI for 48 h. Results are expressed as means ± SEM (n = 3). **p < 0.01, ***p < 0.001 compared with the control group (un-paired *t*-test).

Cell viability using MTT assay was performed to evaluate the tissue-specificity and toxicity of DOI in various cell types, including MCF-7 breast cancer cell line, OVCA-429 ovarian cancer cell line, mouse splenocytes, and rat ovarian granulosa cells. The results showed DOI increased the viability of mouse splenocytes and rat ovarian granulosa cells, but decreased the viability of MCF-7 and OVCA-429 cells in a dose-dependent manner (Fig. 3). The reduction in the viability of normal cells is a key index of toxicity after exposure to a toxic substance. The DOI protein isolated from edible Chinese yam did not cause any apparent toxicity *in vitro*.

### Estradiol-stimulating effect of DOI *in vitro*

**Figure 4:**
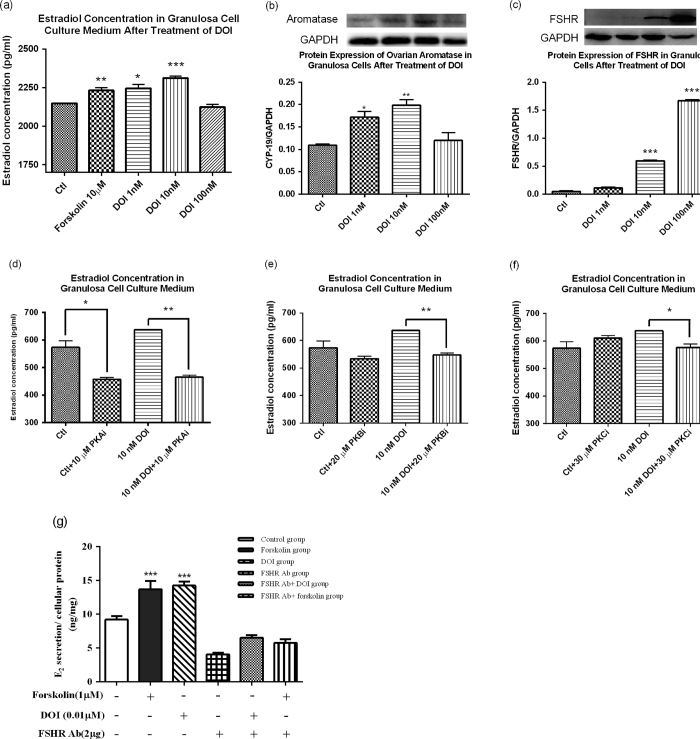
(**a**) Stimulatory activity of DOI on estrogen biosynthesis in granulosa cells. Protein expression of (**b**) aromatase and (**c**) follicle-stimulating hormone receptor (FSHR) in ovarian granulosa cells. Results are expressed as means ± SEM (n = 3). *p < 0.05, **p < 0.01, ***p < 0.001 compared with the control group (un-paired *t*-test). Effects of (**d**) protein kinase A inhibitor (PKAi), (**e**) protein kinase B inhibitor (PKBi), and (**f**) protein kinase C inhibitor (PKCi) on the estrogen-stimulating effect of DOI on ovarian granulosa cells. (**g**) Effects of DOI on FSHR-attenuated ovarian granulosa cells after 12 h treatment. Results are expressed as means ± SEM (n = 3). *p < 0.05, **p < 0.01 compared with the control group (un-paired *t*-test).

To determine the estradiol-stimulating effect of DOI, we measured the estradiol levels in cell culture medium of DOI-treated rat ovarian granulosa. Granulosa cells treated with DOI (1 and 10 nM) had significantly increased estradiol biosynthesis (Fig. 4A) and aromatase expression (Fig. 4b) compared to the control, with Forskolin used as a positive control. DOI (10 nM) had maximal effect on stimulating both estradiol production and aromatase expression, whereas DOI (100 nM) did not show any estrogen-stimulating activity compared with the control. The FSHR expression also increased with DOI (1 to 100 nM) treatments (Fig. 4c).

In the presence of PKA inhibitor (PKA*i*), estradiol levels in the culture medium of granulosa cells were significantly decreased in both the control group and DOI-treated group (Fig. 4d). PKA*i* decreased the DOI (10 nM)-induced estrogen-stimulating activity by one-fold (Fig. 4d). Both PKB and PKC inhibitors (PKB*i* and PKC*i*) significantly decreased estradiol levels in the culture medium of the DOI-treated groups, but did not affect the estradiol levels in the control group (Fig. 4e,f). Our results showed the estradiol-stimulating effect of DOI was abolished in the FSHR-attenuated ovarian granulosa cell model (Fig. 4g).

### Estradiol-stimulating effect of DOI *in vivo*

**Figure 5:**
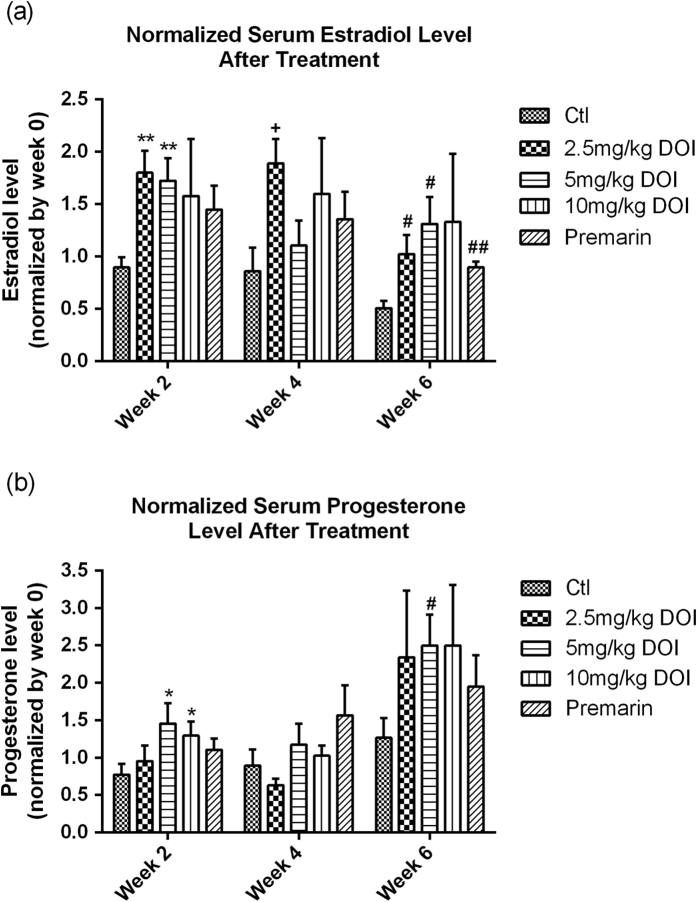
(**a**) Serum estradiol and (**b**) progesterone levels in Sprague Dawley rats after treatment with DOI for 2, 4, and 6 weeks. Results are expressed as means ± SEM (n = 6). *p < 0.05, **p < 0.01 compared with week 2 controls;+p < 0.05 compared with week 4 controls; and ^#^p < 0.05, ^##^p < 0.01 compared with week 6 controls (un-paired *t*-test). Ctl: control group received daily intraperitoneal injections of PBS; Premarin: positive control group received daily Premarin (12.4 mg/kg) by oral administration; DOI groups: DOI-treated groups received daily intraperitoneal injections of DOI (2.5, 5, and 10 mg/kg).

To determine the estradiol-stimulating effect of DOI, we measured the serum estradiol and progesterone levels in our rat model. The levels of estradiol and progesterone reached their peaks after 6 weeks of DOI (5 mg/kg) treatment (Fig. 5). However, treatment with the highest concentration of DOI (10 mg/kg) for 6 weeks did not significantly increase estradiol and progesterone levels compared with the control. Premarin used in HRT also increased estradiol biosynthesis.

The body weights of the Sprague Dawley (SD) rats did not significantly change during the DOI and Premarin treatments (Fig. S1). A reduction in body weight could be a sensitive index of toxicity after the animal has been exposed to a toxic substance. These results indicate that DOI did not cause any apparent toxicity *in vivo*. The ratio of final ovarian weight to final total body weight remained steady after treatment with DOI (2.5 and 5 mg/kg) compared with the control, but treatment with 10 mg/kg DOI and Premarin increased the ovary weight, with the ratio increasing from 0.032% to 0.047% and 0.048% (*p < 0.05*), respectively (Fig. S2).

### Determination of the estrogen-related gene expressions *in vivo*

**Figure 6:**
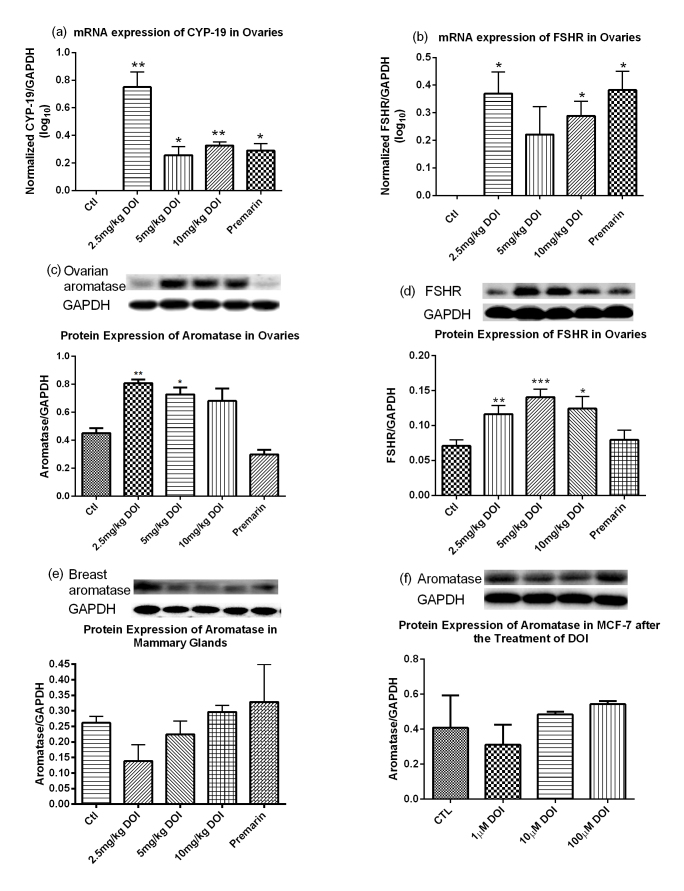
mRNA expression of (**a**) ovarian CYP-19 and (**b**) follicle-stimulating hormone receptor (FSHR), and protein expression of (**c**) ovarian aromatase, (**d**) ovarian FSHR, and (**e**) breast aromatase in Sprague Dawley rats after treatment with DOI for 6 weeks. (**f**) Protein expression of aromatase in DOI-treated MCF-7 breast cancer cell line. Results are expressed as means ± SEM (n = 6). *p < 0.05, **p < 0.01, ***p < 0.001 compared with the control group (One-way ANOVA followed by Dunnett’s Multiple Comparison Test). Ctl: control group received daily intraperitoneal injections of PBS; Premarin: positive control group received daily Premarin (12.4 mg/kg) by oral administration; DOI group: DOI-treated groups received daily intraperitoneal injections of DOI (2.5, 5, and 10 mg/kg).

**Figure 7:**
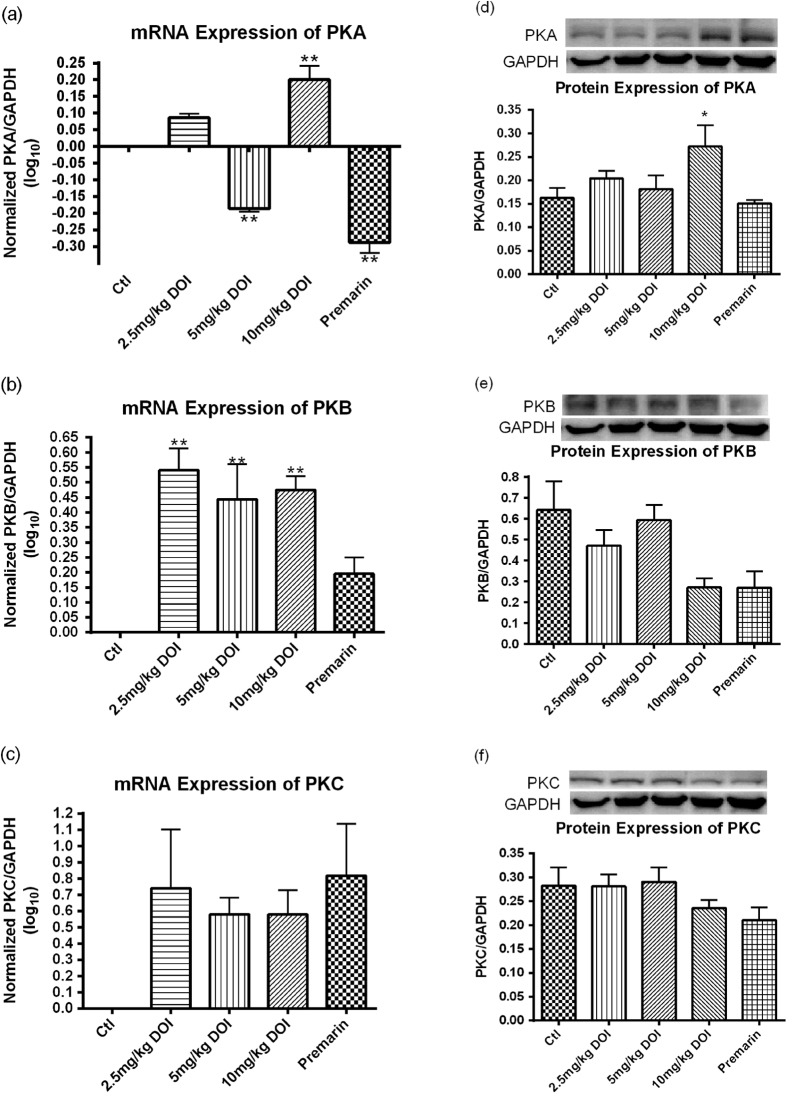
mRNA expression of (**a**) PKA, (**b**) PKB and (**c**) PKC, and protein expression of (**d**) PKA, (**e**) PKB and (**f**) PKC in ovaries of Sprague Dawley rats after treatment with DOI for 6 weeks. Results are expressed as means ± SEM (n = 3 for mRNA; n = 6 for protein). *p < 0.05, **p < 0.01, ***p < 0.001 compared with control group (one-way ANOVA and un-paired *t*-test, respectively).

The estrogen-related gene expression levels in our rat model were determined by real-time PCR. The mRNA expressions of both CYP-19 and FSHR in the ovaries of SD rats were significantly increased after treatment with DOI and Premarin (Fig. 6A,B). The groups treated with 2.5 mg/kg DOI had the highest mRNA expressions among the three DOI dosage groups compared with the control group and the effect was not dose-dependent. The mRNA expressions of both PKA and PKB in the ovaries of SD rats were significantly increased after treatment with DOI (Fig. 7A,B).

### Determination of the estrogen-related protein expressions *in vivo*

The estrogen-related protein expression levels in our rat model were determined by Western blotting analysis. The levels of ovarian aromatase were significantly upregulated in the groups treated with DOI (2.5 and 5 mg/kg), whereas the groups treated with 10 mg/kg DOI had similar levels to the control. The Premarin-treated group had significantly attenuated protein expression level of ovarian aromatase (Fig. 6c,d). The protein expression level of breast aromatase was not upregulated in all DOI treatment groups both *in vitro* and *in vivo* (Fig. 6e,f). The protein expression level of ovarian FSHR was not affected by Premarin treatment, whereas the expression level was significantly enhanced with DOI (2.5, 5, and 10 mg/kg). The protein expression level of PKA was significantly upregulated in the groups treated with 10 mg/kg DOI (Fig. 7D).

### Measurement of bone mineral density and microarchitecture by microCT scanning

**Figure 8:**
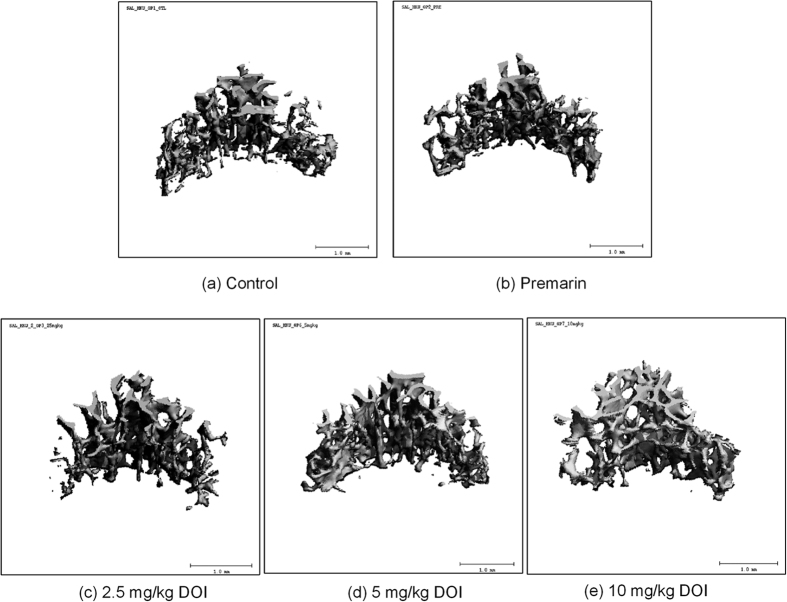
Volume rendered images of L2 in 16- to 18-month-old female SD rats given different treatments (image presented with BV/TV closest to the group mean value).

**Figure 9:**
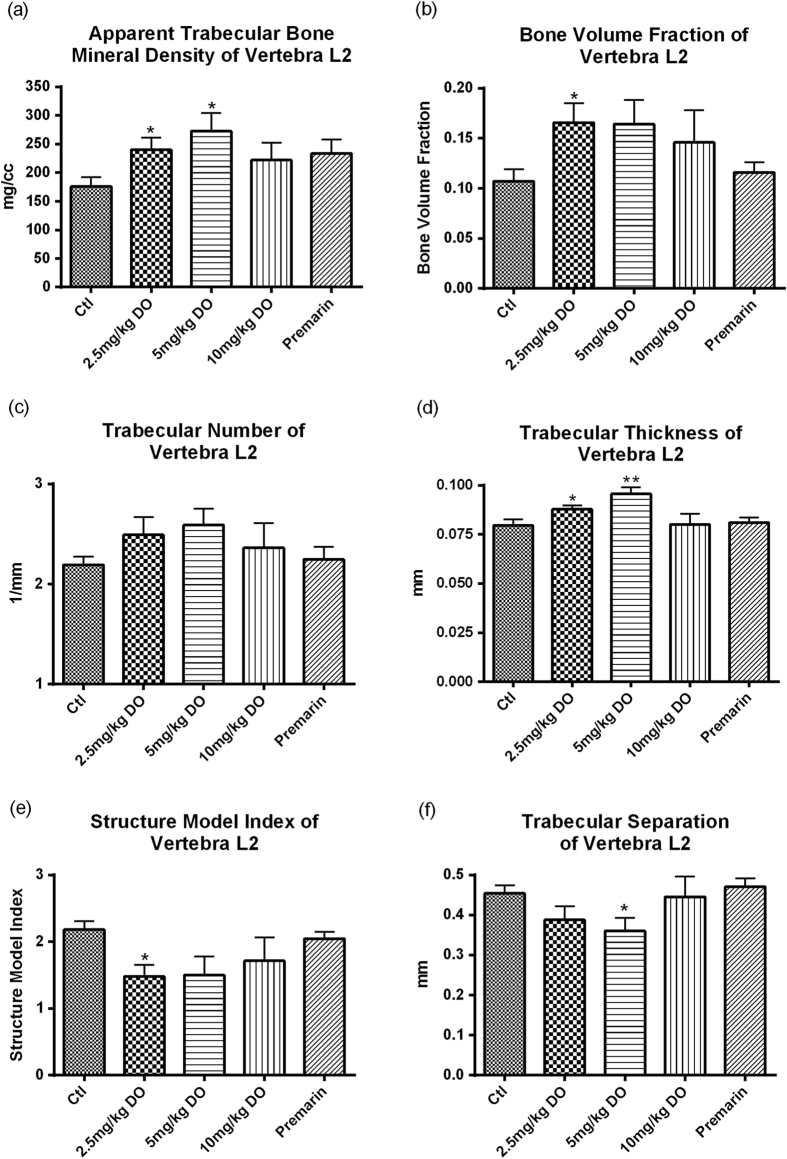
(**a**) Apparent trabecular bone mineral density, (**b**) bone volume fraction, (**c**) trabecular number, (**d**) trabecular thickness, (**e**) structure model index, and (**f**) trabecular separation of vertebra L2 of Sprague Dawley rats after treatment with DOI for 6 weeks. Results are expressed as means ± SEM (n = 6; except Premarin group, where n = 3). *p < 0.05, **p < 0.01 compared with the control group (unpaired *t*-test). Ctl: control group received daily intraperitoneal injections of PBS; Premarin positive control group received daily Premarin (12.4 mg/kg) by oral administration; DOI group: DOI-treated groups received daily intraperitoneal injections of DOI (2.5, 5, and 10 mg/kg).

To determine the osteogenic effects of DOI, we examined the bone mineral density and microarchitecture, which is known to be altered during aging and menopause. Volume rendered images at the 2^nd^ lumbar vertebra (L2) in 18-month-old female SD rats treated with different dosages of DOI are shown in Fig. 8. All DOI treatment groups demonstrated an increase in the apparent bone mineral density of vertebra L2 compared with the control group. The apparent bone mineral density was significantly augmented following DOI (2.5 and 5 mg/kg) treatments (Fig. 9a). DOI, but not Premarin, had a tendency to increase the bone volume fraction of vertebra L2. DOI (2.5 mg/kg) significantly elevated the bone volume fraction compared with the control group (Fig. 9b). The trabecular number of vertebra L2 showed a slight increase after the DOI treatments, but the increase was not significant compared with the control group (Fig. 9c). Treatment with DOI (2.5 and 5 mg/kg) significantly enhanced the trabecular thickness of vertebra L2 compared with the control group (Fig. 9d). All the DOI treatment groups showed a decrease in the structure model index of vertebra L2 compared with the control group, and this reduction was significant in the 2.5 mg/kg DOI treatment group (Fig. 9e). The trabecular separation of vertebra L2 did not show any significant changes following the treatments, with the exception of the 5 mg/kg DOI treatment (Fig. 9f).

### Detection of the protein levels of BDNF and TrkB gp145

**Figure 10:**
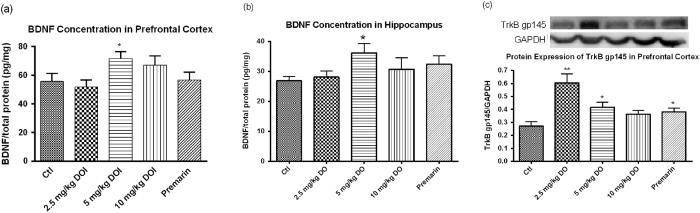
Protein levels of brain derived neurotrophic factor (BDNF) in (**a**) prefrontal cortex and (**b**) hippocampus of Sprague Dawley rats after treatment with DOI for 6 weeks. (**c**) Protein levels of TrkB gp145 receptor in prefrontal cortex of Sprague Dawley rats after treatment with DOI for 6 weeks. Results are expressed as means ± SEM (n = 6). *p < 0.05, **p < 0.01 compared with the control group (un-paired *t*-test). Ctl: control group received daily intraperitoneal injections of PBS; Premarin: positive control group received daily Premarin (12.4 mg/kg) by oral administration; DOI group: DOI-treated groups received daily intraperitoneal injections of DOI (2.5, 5, and 10 mg/kg).

To determine the effects of DOI treatments on cognitive functions, we measured BDNF and its receptor TrkB gp145 in our rat model. The BDNF concentration in the prefrontal cortex was significantly increased after treatment with 5 mg/kg DOI (Fig. 10a,b). The other treatment groups did not show any significant changes. The DOI (2.5 and 5 mg/kg) and Premarin treatments caused significant increases in the expression of TrkB gp145 receptors (Fig. 10c).

## Discussion

Here, we present the first report of a bioactive principle of Chinese yam that can elevate estradiol biosynthesis. We extracted a novel protein, designated as DOI, from *Dioscorea opposita* with a molecular weight of 33.5 kDa as measured by mass spectrometry. We biologically characterized DOI and elucidated its potential pharmacological activity. Two similar proteins of interest, dioscorin^25^ and DJ^23^, have been previously isolated from *Dioscorea batatas* Decne and *Dioscorea japonica,* respectively. In addition, dioscorin has also been isolated from other *Dioscorea* species such as *Dioscorea alata*^26^,^27^. These proteins isolated from yam have different molecular weights and N-terminal sequences, as well as different lectin, antioxidative, immunomodulating, carbonic anhydrase, trypsin inhibiting, chitinase, and estrogen-stimulating activities compared with DOI (Supplementary Table S4).

The stability of proteins can be affected by high temperatures, acid, alkali conditions, enzymatic degradation, and clearance mechanisms. We found that the estrogen-stimulating activity of DOI in ovarian granulosa cells was preserved in DOI treated with HCl (up to 1 M), but this activity was lost with NaOH (0.01–1 M) treatment (Fig. 2a,b). Furthermore, DOI could withstand temperatures up to 80 °C, which significantly increased the activity of DOI (Fig. 2c). Our results indicated that DOI treated with 0.1 M HCl at 80 °C might expose an active site involving a shorter amino acid sequence that has increased activity. These findings could be important for the preparation of more potent DOI-based therapeutics in future studies. DOI itself could be developed into protein therapeutics that exploit its estrogen-stimulating activity. In addition, its half-life and absorption could be prolonged, and its degradation under acidic conditions and high temperatures could be retarded. Nowadays, these issues can be addressed by many formulations and technologies for drug delivery, such as polyethylene glycol (PEG)^28^.

Menopause together with general aging are associated with a decline in immune function. During aging, changes in the immune system such as altered immune tolerance, increase of auto-antibodies, decline in function of natural killer cells, T lymphocytes and B lymphocytes have been reported^29^. Estrogen deficiency after menopause is thought to be related to an increase in the levels of pro-inflammatory serum markers, a reduction in CD4 T lymphocytes and B lymphocytes, and a decrease in the cytotoxic activity of natural killer cells^29^,^30^,^31^. Our *in vitro* study showed that DOI could stimulate the viability of mouse splenocytes (Fig. 3). DOI could be beneficial in preventing the decline in immune functions during menopause.

The gradual decline in the secretion of ovarian estradiol (E_2_) and progesterone (P) are hallmarks of menopause^32^, which also represents the breakdown of normal hypothalamic-pituitary-ovarian (H-P-O) function through loss of feedback regulation of ovarian steroids. Estrogen biosynthesis is catalyzed by aromatase in ovarian granulosa cells^33^. In non-menopausal women, high circulating levels of follicle-stimulating hormone (FSH) induce estrogen secretion by activating the aromatase-encoding gene Cyp19^34^. In aging and menopause, there is a decline in ovarian function and the activity of the ovarian aromatase^35^. Although the circulating level of FSH remains high for several years after menopause, this does not induce the increase of circulating estrogen level. A progressive deterioration in ovarian steroidogenesis appears to be a major factor associated with menopause^35^. Estradiol secretion from ovarian granulosa cells could reveal steroidogenesis and function of the ovary^36^,^37^. Ovarian granulosa cells are often used as a cell model for the evaluation of ovarian steroidogenesis^36^,^37^. FSH is important for granulosa cell steroidogenesis by increasing the expression and activity of aromatase resulting in increased biosynthesis of estradiol^38^. In the classically described mechanism of the function of FSHR, ligand binding results in the activation of adenylate cyclase, increased production of cAMP, and activation of PKA and PKB^39^,^40^,^41^, which then directly influences the expression and activation of aromatase and PKC as a negative regulator^42^.

The results from our *in vitro* study demonstrated that DOI treatment could upregulate FSHR and aromatase expression in ovarian granulosa cells, indicating the increased estradiol biosynthesis could be via the FSHR-aromatase pathway (Fig. 4a–c). Results of the protein kinase inhibition assay suggested that PKA, PKB, and PKC were involved, because the activity of DOI was abolished by inhibitors of PKA, PKB, and PKC (Fig. 4d,e). Furthermore, the estradiol-stimulating effect of DOI was abolished in the FSHR-attenuated ovarian granulosa cell model, confirming DOI promoted estradiol biosynthesis, at least, via interaction with FSHR (Fig. 4g).

A progressive deterioration in the neuroendocrine axis, including ovarian steroidogenesis, in female rats and women appears to be a major factor associated with typical reproductive aging^43^,^44^,^45^,^46^,^47^,^48^. To mimic the reproductive failure in menopausal women, we used 16- to 18-month-old female SD rats with low serum estrogen levels as the animal model, because the gradual decline of neuroendocrine function in rats is similar to humans. DOI could delay the onset of estrogen deficiency associated with aging when compared with their corresponding control groups (Fig. 5a). DOI induced an increase in serum estradiol and progesterone levels *in vivo* after treatment for 6 weeks in a dose-dependent manner. Peak levels of estradiol and progesterone occurred with a concentration of 5 mg/kg DOI, whereas the levels declined with a concentration of 10 mg/kg DOI (Fig. 5). This bell-shaped dose-response curve was found, especially for the stimulation of progesterone. Our *in vivo* experiments revealed the underlying mechanism for the increase in the estradiol levels could be via the upregulation of protein levels of FSHR, aromatase (Fig. 6), and PKA (Fig. 7d). This possible pathway for the action of DOI involving FSHR, PKA, and aromatase is also a classical pathway for steroidogenesis. Data from the Western blot analysis revealed that DOI (2.5 and 5 mg/kg) treatment upregulated aromatase in the ovary (Fig. 6c), but not in breast tissue (Fig. 6e) or MCF-7 breast cancer cells (Fig. 7f). In addition, DOI decreased the viability of MCF-7 breast cancer cells and OVCA-429 ovarian cancer cells in a dose-dependent manner. Thus, estrogen-stimulating activity of DOI is not expected to display any of the side effects of hormone replacement therapy such as the increased risk of breast and ovarian carcinogenesis.

Osteoporosis is one of the major menopausal symptoms, which is characterized by bone mass reduction and skeletal microarchitectural deterioration, resulting in an increased risk of fractures. The loss of ovarian hormones (estrogen and progesterone) during menopause is one of the major risk factors for osteoporosis, which causes increased bone resorption and decreased bone formation^49^,^50^ Bone remodeling increases substantially in the years after menopause, which contributes to age-related skeletal fragility in women^51^. It has been shown that trabecular bone mineral density, trabecular bone volume fraction, trabecular thickness, and trabecular number all decrease with age^52^. Also, a lower bone mass is primarily characterized by a smaller plate-to-rod ratio^53^. All these changes would weaken bone strength and increase the risk of osteoporotic fracture. The trabecular bone mineral density at the L2 mid-vertebral body has been used for the clinical assessment of osteoporosis^54^, and we used this to evaluate the anti-osteoporotic effect of DOI in our study. We found that DOI had beneficial effects on the bone mineral density and microarchitecture *in vivo*. We observed increased bone mineral density, bone volume fraction, trabecular number and trabecular thickness, and decreased structure model index and trabecular separation of the vertebra L2 using high resolution microCT (Fig. 9a–f). These changes in bone density and microarchitecture favor the enhancement of bone strength^55^,^56^. The decrease in structure model index indicated an increase in the ratio of plate-shaped to rod-shaped trabeculae. The latter trabecular structure is dominant in osteoporosis^57^. The increase in plate-shaped trabeculae found in the present investigation indicated the bone may be less porous as reflected in the parallel decrease of trabecular separation. This suggests DOI could exert an anti-osteoporotic effect on the trabecular bone *in vivo*. Together with the effects on estradiol and progesterone biosynthesis, DOI might be a potential therapeutic agent for menopausal osteoporosis.

Cognitive decline is a common phenomenon experienced by menopausal women^58^. A recent study demonstrated that plasma BDNF level can be used as a biomarker for cognitive function in aging women^59^. Another study found BDNF was significantly decreased in older human subjects^60^. Administration of exogenous BDNF was found to offset some physiological or pathological age-associated changes in the central nervous system^61^. BDNF is widely expressed in the brain. It belongs to the neurotrophin family, which is a group of small basic secreted proteins that aid in the survival, maintenance, and plasticity of specific neuronal populations. In this study, treatment with DOI increased the protein expression levels of BDNF in the prefrontal cortex and hippocampus, and TrkB gp145 in the prefrontal cortex (Fig. 10). Thus, DOI could have beneficial effects because the prefrontal cortex is important for cognitive function^62^,^63^. However, the pharmacological effect might not be directly due to the DOI protein, as it cannot cross the blood brain barrier to reach the prefrontal cortex. The effect might be due to the increased serum estradiol levels, because it is thought that estradiol is neuroprotective and can increase the expression of BDNF^64^,^65^.

The exact mechanism of how DOI stimulates the biosynthesis of estradiol requires further investigation. Our data suggest that DOI might act on ovarian aromatase, but whether the upregulation of aromatase is a direct effect of DOI remains unknown. Therefore, the effect of aromatase inhibitor on the DOI-treated granulosa cells will need to be studied, which would help to confirm our proposed mechanism. Further studies on the stimulating effect of DOI on estradiol secretion using the ovariectomized SD rat model^66^ will help to elucidate whether the increased serum estradiol level was due to increased ovarian estradiol biosynthesis or was from other organs. In this study, the bell-shaped dose-response curves of the effects of DOI on estrogen biosynthesis *in vitro* (Fig. 4A) and *in vivo* (Fig. 5A) were measured. Further study with a wider range of DOI concentrations will be required to provide information about the most effective dosage of DOI.

## Conclusion

The novel DOI protein isolated from *Dioscorea opposita* stimulated estradiol secretion by binding to FSHR, and upregulated the expression of ovarian FSHR and aromatase both *in vitro* and *in vivo* predominantly through the activation of PKA. DOI also induced the secretion of estradiol and progesterone in aged female SD rats. More importantly, DOI exhibited tissue-specific bioactivity as it enhanced the proliferation of splenocytes and ovarian granulosa cells, but did not stimulate the proliferation of breast cancer cells and ovarian cancer cells. Furthermore, DOI could upregulate protein expression levels of ovarian aromatase but not breast aromatase *in vitro* and *in vivo*. These results suggest that DOI could be a more efficacious and safer alternative to HRT for the treatment of menopausal syndrome. Apart from improving the hormonal status, DOI could be beneficial for menopausal osteoporosis and improve cognitive functioning. Findings from this study have been used to apply for patents (Patent Cooperation Treaty Patent Pub. No.: WO/2013/023617; U.S. Patent Pub. No.: US 20130217626 A1; European Patent Pub. No.: EP 2750684 A1; China Patent pub. No.: CN 103945854 A), and we are seeking industrial partners for further research and future development of DOI as the first protein drug for the treatment of menopausal syndrome.

## Methods

### Protein extraction

Rhizomes of *Dioscorea opposita* were obtained from a market on Centre Street in Hong Kong. The tubers were authenticated by Dr Yanbo Zhang, one of the authors of this manuscript. The rhizomes were peeled and homogenized in an extraction buffer (5% acetic acid, 0.1% β-mercaptoethanol) in a ratio of 1:2 (w/v) for 3 h at 4 °C. The homogenate was centrifuged at 17700 g for 30 min at 4 °C. Ammonium sulfate was added to the supernatant to 80% saturation. The mixture was stirred at 4 °C overnight and centrifuged at 17700 g for 1 h at 4 °C. The precipitated proteins were resuspended in Milli-Q H_2_O. The protein extract was dialyzed against double-distilled H_2_O overnight and then ultra-centrifuged at 40000 g for 2 h at 4 °C. The supernatant was collected for further purification by FPLC.

### Column purification of DOI protein

The supernatant was adjusted to a final concentration of 100 mM Tris (pH 8.0) and was loaded on a HiPrep 16/10 DEAE FF column (GE Healthcare, Sweden) for purification using an AKTA Purifier (GE Healthcare, Sweden) FPLC system (Buffer A: 100 mM Tris, pH 8.0; Buffer B: 1 M NaCl, with 100 mM Tris, pH 8.0. Gradient 0% – 45% Buffer B). Fraction D3 was collected and dialyzed against double-distilled H_2_O overnight. Fraction D3 (Fig. 1a) was adjusted to 50% Buffer B and loaded on a HiPrep 16/10 Phenyl FF (high sub) column (GE Healthcare, Sweden) for FPLC (Buffer A: Milli-Q H_2_O; Buffer B: 10 mM sodium phosphate, pH 7.0 with 1 M (NH_4_)_2_SO_4_. Gradient: 30% – 0% Buffer B). Fraction P1 (Fig. 1b) was collected and dialyzed against double-distilled H_2_O overnight and then lyophilized into powder form. The lyophilized powder was dissolved in 50 mM sodium phosphate (pH 7.2) containing 150 mM NaCl and then loaded on a Superdex 75 10/300 GL column (GE Healthcare, Sweden) for FPLC (Buffer A: 50 mM sodium phosphate, pH 7.2 with 150 mM NaCl. Flow rate = 0.6 mL/min). The column was calibrated with the above buffer using molecular mass markers: aprotinin (6500 Da), ovalbumin (44287 Da), and bovine serum albumin (66000 Da). Fraction S1 containing the DOI protein (Fig. 1c) was collected and dialyzed against double-distilled H_2_O overnight and then lyophilized into powder form and stored at −20 °C for the biological characterization.

### Chemical characterization of DOI

The molecular weight of DOI was determined by size-exclusion chromatography on a Superdex 75 10/300 GL column (GE Healthcare, Sweden), by mass spectrometry, and by 15% Native PAGE and 15% SDS-PAGE with silver staining. The N-terminal sequence was determined by Edman degradation using a 494 Procise Protein Sequencer/140C Analyzer (Applied Biosystems, Inc., USA) with the assistance of the Protein Facility of Iowa State University, USA. The N-terminal sequence was searched with BLAST. The partial amino acid sequence was measured by mass spectrometry with the support of the Proteomics Centre in the VU University, Amsterdam, the Netherlands. DOI (4.5 μg) was redissolved in 50 mM ammonium bicarbonate and incubated with 300 ng trypsin/Lys-C Mix (Mass Spec grade; Promega, USA) at 37 °C for 16 h, and then dried in a speedvac. Peptides were analyzed by nanoLC-MS/MS using an Ultimate 3000 LC system (Dionex, Thermo Scientific, CA, USA) coupled to the TripleTop 5600 mass spectrometer (Sciex, Ontario, Canada). Peptides were trapped on a PepMap 100 C18 column (5 mm × 300 μm ID, 5-μm particle size; Dionex) and fractionated on a Alltima C18 column (200 mm × 100 μm ID, 3-μm particle size; Thermo Fisher Scientific, MA, USA). The acetonitrile concentration in the mobile phase was increased from 5% to 40% in 30 min and to 90% in 3 min at a flow rate of 400 nL/min. The eluted peptides were electrosprayed into the TripleTop MS. The mass spectrometer was operated in a data-dependent mode with a single MS full scan (m/z 350-1200) followed by top 20 MS/MS scan. The data were searched with MaxQuant using the UniProt/SwissProt plant database.

### Biological characterization of DOI

#### Determination of estradiol-stimulating effect of DOI by in vitro estrogenic assay

Nine female Sprague Dawley rats (21 to 23 days old; 3 rats per group) were primed with 80 IU pregnant mare serum gonadotropin (PMSG; Sigma-Aldrich, USA) for 48 h to stimulate follicular development. The rats were sacrificed and their ovaries were collected. The ovarian follicles were punctured with a 25-gauge needle to isolate granulosa cells. Cells in serum-free DME/F12 1:1 medium supplemented with 1% penicillin-streptomycin and 1% bovine serum albumin were seeded at a density of 1 × 10^5^ cells per well in a 96-well plate and cultured for 2 h at 37 °C in an atmosphere of 5% CO_2_. The ovarian granulosa cells were then treated with DOI (1, 10, 100 nM)and incubated for 12 h. The cell culture medium was collected for the measurement of estradiol concentrations and cellular proteins were extracted for Western blot analysis. The experiments were approved by the Committee on the Use of Live Animals in Teaching and Research (CULATR 1824-09) of Li Ka Shing Faculty of Medicine, The University of Hong Kong.

#### Determination of acid, alkali and thermal stability, and chitinase activity of DOI

For the acid and alkali stability tests, DOI (10 nM) was treated with HCl (0.01, 0.1, and 1 M) or NaOH (0.01, 0.1, and 1 M) and incubated at 4 °C for 30 min to determine acid stability and alkali stability, respectively. The mixtures were then neutralized with either NaOH or HCl. The acid- or alkali-treated DOI was then added to the granulosa cells and incubated for 12 h. The cell culture medium was collected for the measurement of estradiol concentrations. For the thermal stability test, DOI was incubated at 60 °C, 80 °C, or 100 °C for 30 min. The heat-treated DOI was then added to the granulosa cells and incubated for 12 h. The cell culture medium was collected for the measurement of estrogen concentrations. The chitinase activity of DOI was measured using a Chitinase Assay Kit (# CS0980, Sigma-Aldrich) following the manufacturer’s instructions. Three substrates, 4-nitrophenyl N,N′-diacetyl-β-D-chitobioside (N6133), 4-nitrophenylN-acetyl-β-D-glucosaminide (9376), and 4-nitrophenyl β-D-N,N′,N′′-triacetylchitotriose (N8638) were used to evaluate the exochitinase activity (chitobiosidase activity), exochitinase activity (β-N-acetylglucosaminidase activity), and endochitinase activity of DOI, respectively.

#### Evaluation of the viability of MCF-7 cells, OVCA-429 cells, mouse splenocytes, and rat ovarian granulosa cells after incubation with DOI by MTT assay

MCF-7 estrogen receptor positive-breast cancer cells and OVCA-429 cancer cells with estrogen receptor were seeded at a density of 3 × 10^4^ and 1.5 × 10^4^ cells per well in 96-well microplates, respectively. They were serum starved for 24 h prior to drug treatment. Mouse splenocytes, isolated from BALB/c mice, were diluted with RPMI 1640 medium containing 15% fetal bovine serum and 1% penicillin-streptomycin and seeded at a density of 5 × 10^5^ cells in 100 μL culture medium per well in 96-well microplates. The ovarian granulosa cells were prepared as described above. All cells were incubated at 37 °C in a humidified atmosphere with 5% CO_2_ for 24 h. For the granulosa cells and cancer cells, DOI in complete medium at a final concentration of 1, 10, and 100 nM was added and incubated for 48 h, whereas DOI in RPMI 1640 medium with 15% fetal bovine serum and 1% penicillin-streptomycin was added to the mouse splenocytes and incubated for 72 h. The cells were then incubated with 10 μL MTT solution (5 mg/mL) for 3 h. The formazan crystals were dissolved in DMSO and absorbance at O.D. 540 nm was measured using a microplate reader (Model 680; Bio-Rad, Hercules, California, USA). Percentage viability relative to the control was calculated.

### Estradiol-stimulating effect of DOI *in vitro*

DOI (1, 10, 100 nM) was added to the ovarian granulosa cells and incubated for 12 h. The cell culture medium was collected for the measurement of estrogen concentrations and cellular proteins were extracted for Western blot analysis. Western blotting was performed on proteins from ovarian granulosa cells using specific anti-FSHR (sc-13935; Santa Cruz Biotechnology, Inc, USA) and anti-aromatase (sc-14245; Santa Cruz Biotechnology, Inc) antibodies. For the kinase inhibition assay, granulosa cells in serum-free DME/F12 1:1 medium supplemented with 1% penicillin-streptomycin and 1% bovine serum albumin were seeded at a density of 1 × 10^6^ cells in a 24-well plate and were incubated at 37 °C in an atmosphere of 5% CO_2_ for 1 h. The cells were then treated with either 10 μM protein kinase A inhibitor (H-89), 20 μM protein kinase B inhibitor (LY-294002), or 30 μM protein kinase C inhibitor (GF-109203X) for 1 h. DOI (10 nM) was then added and the cells were incubated for another 12 h. The cell culture medium was collected for the measurement of estrogen concentrations to evaluate the role of PKA, PKB, and PKC. In addition, to identify whether DOI binds to FSHR in an FSHR-attenuated ovarian granulosa cell model, ovarian granulosa cells were pretreated with 2 μg of FSHR antibody (sc-7798, Santa Cruz Biotechnology, Inc) as an FSHR antagonist for 30 min. The cells were then treated with DOI (0.1 and 0.01 μM), 1 μM Forskolin (Sigma-Aldrich) as a positive control, or 2 μg of FSHR antibody as a negative control for 12 h. The cell culture medium was collected for the measurement of estradiol concentrations.

### Estradiol-stimulating effect of DOI *in vivo*

#### Animal model

All experiments were performed in accordance with relevant guidelines and regulations of the Committee on the Use of Live Animals in Teaching and Research (CULATR 1824-09) of Li Ka Shing Faculty of Medicine, The University of Hong Kong. Female SD rats (16 to 20 months old) with low serum estrogen levels were used as an animal model for aging. Female SD rats (8 months old) from the Laboratory Animal Unit, The University of Hong Kong, were maintained until they were 16 to 20 months old. The animals were housed in an air-conditioned room at an ambient temperature of 24 °C and relative humidity of 50%–65% under automatic 12-h light/12-h dark cycles.

#### Drug administration and collection of serum and organs

Female SD rats (16 to 20 months old) were randomly divided into six groups (n = 6). Group 1 was the control group given intraperitoneal injections of phosphate buffered saline (PBS) only; Groups 2, 3, and 4 were given intraperitoneal injections of DOI at three different dosages (2.5, 5, and 10 mg/kg), respectively; and Group 5 received an oral administration of Premarin (12.4 mg/kg). Each rat was treated daily for 6 weeks. Blood samples were collected from the tail vein once every 2 weeks for the measurement of serum estrogen and progesterone levels. At the end of the experiment, the ovaries, the 1^st^ to 6^th^ lumbar vertebrae, brains, and breast tissues were collected and stored at −80 °C for further analysis. Their final body weight and final ovarian weight were also measured.

#### Detection of hormone levels

Estradiol and progesterone levels were measured using commercially available electro-chemiluminescence immunoassay (ECLIA) kits (Estradiol II #03000079 and Progesterone II #12145383; Roche Diagnostics, IN, USA) on a Elecsys 2010 autoanalyzer (Roche Diagnostics) following the manufacturer’s instructions.

#### Determination of estrogen-related gene expression by real time PCR

Total RNA was extracted from rat ovarian granulosa cells and ovaries using the High Pure RNA Isolation Kit (Roche Applied Science) following the manufacturer’s instructions. cDNA was synthesized from total RNA using the First Strand cDNA Synthesis Kit (Fermentas) following the manufacturer’s instructions. Real time PCR was performed using a LightCycler 480 Real-Time PCR system (Roche Applied Science). Primers were as follows: ovarian CYP-19 (Forward: 5′ GAGAGTTCATGAGAGTCTGGATCA, Reverse: 5′ GATATAGTTGCTGTGCTTCATCA), ovarian FSHR (Forward: 5′ GAAAGGATCATTTGCTGGATTT, Reverse: 5′ CTTCCAAGACATCATTCTGAGAGA), ovarian PKA (Forward: 5′ TGGATGTGATCGGGGAAA, Reverse: 5′ AAGCTGTCGGCCTTTTCA), ovarian PKB (Forward: 5′ AAAACTTTCTTCGTCCACACG, Reverse: 5′ GGACTGCTCTGGTACTGTTGC), and ovarian PKC (Forward: 5′ GCATAGACTGGGACCTGCTT, Reverse: 5′ CCAGGCCATAGTCATCTGTG).

#### Determination of estrogen-related proteins by Western blot analysis

Proteins were extracted from rat ovarian granulosa cells, ovaries and breast tissues, and MCF-7 breast cancer cells using RIPA buffer (Sigma-Aldrich) with complete protease inhibitor cocktail tablets (Roche Applied Science). Denatured proteins (20 μg per sample) were separated on SDS-PAGE and transferred to PVDF membranes. For proteins from ovarian granulosa cells and ovaries, immunoblotting was performed using specific anti-FSHR (sc-13935; Santa Cruz Biotechnology, Inc), anti-aromatase (sc-14245; Santa Cruz Biotechnology, Inc), anti-phospho-PKA (#04-404; Upstate, Millipore), anti-p-AKT1/2/3 (ser 473)-R (sc-7985-R; Santa Cruz Biotechnology, Inc) and anti-phospho-PKC (Thr555/Thr563; Upstate, Millipore) antibodies with anti-GAPDH antibody as the internal standard. After incubation with horseradish peroxidase-conjugated secondary antibody, the chemiluminescence (GE Bio-health) was detected with the Bio-Rad Chemi Doc™ EQ densitometer (Bio-Rad) and quantified by Bio-Rad Quantity One 1-D Analysis software (Bio-Rad).

#### Measurement of bone mineral density and microarchitecture by microCT scanning

Sprague Dawley rats were treated with DOI for 6 weeks. Lumbar vertebrae including soft tissue were harvested and wrapped in normal saline gauze (0.9%) and stored at −80 °C until use. Bone status was evaluated using an In Viva MicroCT 40 computed tomography system (Scanco Medical, Bassersdorf, Switzerland). For image acquisition, the thawed lumbar vertebrae specimens wrapped in saline gauze were placed in a sample holder and the long axis was aligned with the axis of rotation of the X-ray gantry. A scout view was obtained, which was used to identify the vertebra L2 to determine the scanning location. A set of 100 contiguous axial slices at an isotropic resolution of 21 μm was prescribed at the L2 mid-vertebral body. Exposure parameters were set at X-ray tube peak voltage of 70 kVp, tube current of 114 μA, and integration time of 300 ms. Calibration of X-ray attenuation to bone mineral density (mg/cc of hydroxyapatite, HA) on the microCT scanner was carried out weekly using a standardized phantom. For Image processing and analysis, a Gaussian filter (support = 2 and sigma = 1.2) was used to reduce the image noise. Precise contouring was performed in each of the slices by manually drawing the region of interest a few voxels away from the endocortical boundary. This was then followed by segmentation using a global threshold of 250 for all the study groups to separate the mineralized tissues from non-mineralized tissues. Volume rendering of the segmented slices was performed to provide a three-dimensional (3D) image. The apparent trabecular bone mineral density and its bone microarchitecture were automatically evaluated using the built-in program of the microCT system using the model-independent direct 3D morphometry. The bone microarchitecture including apparent trabecular bone mineral density, bone volume fraction, trabecular number, trabecular thickness, trabecular separation, and structural model index were determined (see Supplementary Table S5 for an explanation of the bone microarchitecture parameters)^67^.

#### Detection of the protein levels of BDNF and TrkB gp145

The protein levels of BDNF in rat prefrontal cortex and hippocampus were measured using a BDNF Sandwich ELISA Kit (#CYT306; Millipore) following the manufacturer’s instructions. The protein expression levels of BDNF were normalized using the total protein concentration of the individual samples. Immunoblotting was performed using anti-TrkB receptor antibody (sc-8316; Santa Cruz Biotechnology, Inc) for proteins from the prefrontal cortex with anti-GAPDH antibody as the internal standard. After incubation with horseradish peroxidase-conjugated secondary antibody, the chemiluminescence (GE Bio-health) was detected using a Bio-Rad Chemi Doc™ EQ densitometer (Bio-Rad) and quantified by the Bio-Rad Quantity One 1-D Analysis software (Bio-Rad).

## Additional Information

**How to cite this article**: Lok K. WONG *et al*. A Novel, Stable, Estradiol-Stimulating, Osteogenic Yam Protein with Potential for the Treatment of Menopausal Syndrome. *Sci. Rep.* **5**, 10179; doi: 10.1038/srep10179 (2015).

## Supplementary Material

Supporting Information
