# Erratum: A Novel, Stable, Estradiol-Stimulating, Osteogenic Yam Protein with Potential for the Treatment of Menopausal Syndrome

**DOI:** 10.1038/srep17129

**Published:** 2015-12-18

**Authors:** Kam Lok Wong, Yau Ming Lai, Ka Wan Li, Kai Fai Lee, Tzi Bun Ng, Ho Pan Cheung, Yan Bo Zhang, Lixing Lao, Ricky Ngok-Shun Wong, Pang Chui Shaw, Jack Ho Wong, Zhang-Jin Zhang, Jenny Ka Wing Lam, Wen-cai Ye, Stephen Cho Wing Sze

Scientific Reports 5 Article number: 10179; 10.1038/srep10179 published online: 07102015; updated: 12182015.

The original version of this Article contained an error in the spelling of the author Wen-cai Ye, which was incorrectly given as Ye Wencai.This has now been corrected in the PDF and HTML versions of the Article.

