# Supplementary material for: A Novel, Stable, Estradiol-Stimulating, Osteogenic Yam Protein with Potential for the Treatment of Menopausal Syndrome
Source: Sci Rep. 2015 Jul 10;5:10179. doi: 10.1038/srep10179 (PMC5155516; doi:10.1038/srep10179)
Supplement: Supporting Information [file srep10179-s1.pdf]

<u>**SUPPLEMENTARY INFORMATION**</u>

A Novel, Stable, Estradiol-Stimulating, Osteogenic Yam Protein with Potential for the Treatment of Menopausal Syndrome

Kam Lok WONG[1], Yau Ming LAI[2], Ka Wan LI[3], Kai Fai LEE[4], Tzi Bun NG[5], Ho Pan CHEUNG[1], Yan Bo ZHANG[1], Lixing LAO[1], Ricky Ngok-Shun WONG[6], Pang Chui SHAW[7], Jack Ho WONG[1,5], Zhang Jin ZHANG[1], Jenny Ka Wing LAM[8], Wencai YE[9] & Stephen Cho Wing SZE[1,*]

TABLE S1. Summary of the purification of DOI.

Purification steps	Total protein (mg)/100 g *Dioscorea opposita*	Protein yield (%)
Homogenate	165.19	100
Supernatant after ammonium sulfate precipitation, dialysis, and ultracentrifugation	72.96	44.17
fraction D3	13.12	7.94
fraction P1	6.48	3.93
fraction S1 (DOI)	0.60	0.36

TABLE S2. Calculation of the molecular weight of DOI (32.5 kDa) by size-exclusion chromatography.

Superdex 75 10/300 GL				
Bed volume (ml)	18.8			
Void volume (ml)	7.5			
Sample/marker	Retention volume (ml)	Kav	Molecular weight (MW, kDa)	log MW
Bovine serum albumin	9.35	0.16	66.0	4.82
Ovalbumin	10.12	0.23	44.3	4.66
Aprotinin	15.25	0.69	6.5	3.81
DOI	11.06	0.31	32.5	4.51

TABLE S3. BLAST analysis of the N-terminal sequence of DOI. A high E value ($>10^{-3}$) indicates the N-terminal sequence is novel.

Protein	Residue no.	Sequence	Residue no.	E-value
DOI	1	GIGKITTYWGQYSDEPSLTEA	21	/
allergen Ziz m 1 [*Ziziphus mauritiana*] AAX40948.1	25	Query 2 IGKITTYWGQY--SDEPSLTEA 21<br>+G I TYWGQY +E SL EA<br>Sbjct 25 VGGIATYWGQYTETEEGSLAEA 46	46	0.028
class III chitinase [*Medicago truncatula*] AAQ21404.1	28	Query 3 GKITTYWGQYSDEPSLTEA 21<br>GKI YWGQ +E L EA<br>Sbjct 28 GKISIYWGQNGNEGTLAEA 46	46	0.31
putative secreted hydrolase [*Pseudoalteromonas tunicata* D2] ZP 01135667.1	691	Query 1 GIGKITTYWGQ------YSDEPS 17<br>GI IT YWGQ YS E S<br>Sbjct 691 GISNITSYWGQGWWGYLYSGEAS 713	713	0.43
msrD protein [*Nocardia seriolae*] BAI22688.1	100	Query 3 GKITTYWGQYSD 14<br>GKIT YWG YSD<br>Sbjct 100 GKITEYWGNYSD 111	111	0.6
chitinase-like xylanase inhibitor protein [*Coffea arabica*] ADZ48381.1	4	Query 5 ITTYWGQYSDEPSLTEA 21<br>I TYWGQ DE SL +A<br>Sbjct 4 IATYWGQNTDEGSLEDA 20	20	0.61
pathogenesis-related protein 8 [*Malus x domestica*] ABC47924.1	29	Query 5 ITTYWGQYSDEPSLTEA 21<br>I TYWGQ +E L EA<br>Sbjct 29 IATYWGQNGNEGTLAEA 45	45	1.7
acidic class III chitinase [*Rehmannia glutinosa*] AAO47731.1	25	Query 3 GKITTYWGQYSDEPSLTE 20<br>GKI YWGQ +E L E<br>Sbjct 25 GKISIYWGQNGNEGTLAE 42	42	2.4
transcriptional regulator, AraC family [*Frankia* sp. EUN1f] ZP 06413816.1	121	Query 6 TTYWGQYSDE 15<br>TTYW QYSDE<br>Sbjct 121 TTYW-QYSDE 129	129	3.4

TABLE S4. Comparison of the characteristics of proteins isolated from different *Dioscorea* species. The estrogen-stimulating activity of proteins derived from *Dioscorea* species has not yet been reported in any study.

	DOI (Our study)	Dioscorin[25-27]	DJ[23]
Species	*Dioscorea opposita* Thunb.	*Dioscorea batatas* Decne/ *Dioscorea alata cv*	*Dioscorea japonica*
Lectin activity	No	Yes	N/A
Antioxidative activity	N/A	Yes	N/A
Immunomodulating/ Immunostimulating activity	Yes	Yes	N/A
Carbonic anhydrase activity	N/A	Yes	N/A
Trypsin inhibiting activity	N/A	Yes	N/A
N-terminal sequence/ partial amino acid sequence	Yes (E value of N-terminal > 10^{-3}, which is novel)	Yes	Yes
Chitinase activity	No	N/A	Yes
Estradiol-stimulating activity	Yes	N/A	N/A
Molecular weight	33.5 kDa*	31 kDa (Sub-unit)	28 kDa

*by mass spectrometry

TABLE S5. List of bone microarchitecture parameters used to evaluate bone mineralization and to measure anti-osteoporotic activity[67]

Abbreviation	Variable	Description	Standard unit
tBMD ↑	Apparent trabecular bone mineral density	Bone mineral density of the total bone volume including the bone marrow	mg/cc of HA
BV/TV ↑	Bone volume fraction	Ratio of the segmented bone volume to the total volume of the region of interest	%
Tb.N ↑	Trabecular number	Measure of the average number of trabeculae per unit length	1/mm
Tb.Th ↑	Trabecular thickness	Mean thickness of trabeculae assessed using direct 3D methods	mm
Tb.Sp ↓	Trabecular separation	Mean distance between trabeculae assessed using direct 3D methods	mm
SMI ↓	Structure model index	An indicator of trabecular structure; SMI = 0 for parallel plates and 3 for cylindrical rods	dimensionless

FIGURE S1.

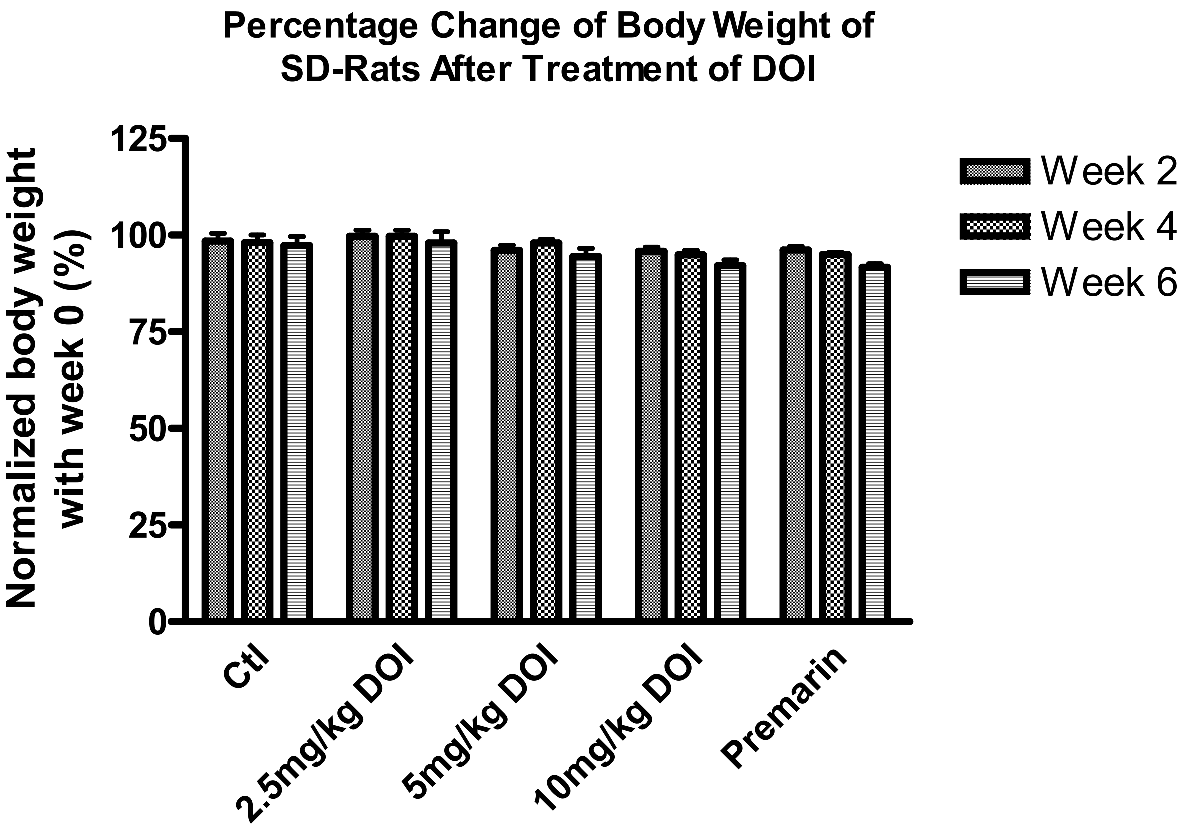

FIGURE S1. Percentage change of body weight in Sprague–Dawley rats after treatment with DOI for 2, 4, and 6 weeks. Results are expressed as means ± SEM, n=6. Ctl: control group received daily intraperitoneal injections of PBS; Premarin: positive control group received daily Premarin (12.4 mg/kg) by oral administration; DOI groups: DOI-treated groups received daily intraperitoneal injections of DOI (2.5, 5, and 10 mg/kg). No statistical significant differences were found among different groups (un-paired *t*-test).

.

FIGURE S2.

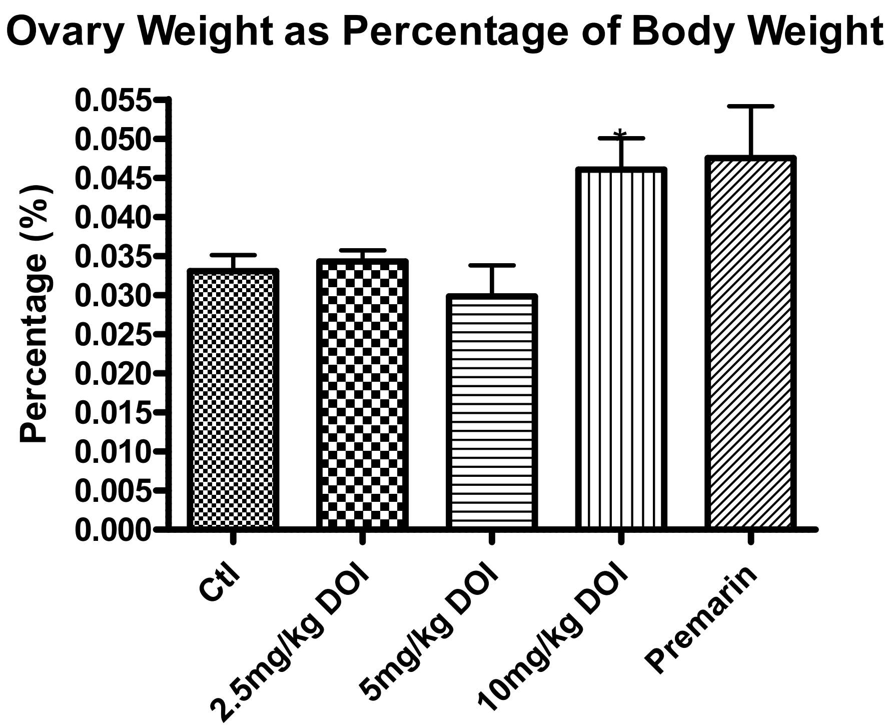

FIGURE S2. Ovary weight over body weight of aged female SD-rat after treatment with DOI for 6 weeks. Results are expressed as means ± SEM, n=6. $^*p < 0.05$ compared with week 6 controls (un-paired *t*-test). Ctl: control group received daily intraperitoneal injections of PBS; Premarin: positive control group received daily Premarin (12.4 mg/kg) by oral administration; DOI groups: DOI-treated groups received daily intraperitoneal injections of DOI (2.5, 5, and 10 mg/kg).
